# Socioeconomic determinants of nutritional status among ‘Baiga’ tribal children In Balaghat district of Madhya Pradesh: A qualitative study

**DOI:** 10.1371/journal.pone.0225119

**Published:** 2019-11-21

**Authors:** Shirisha P.

**Affiliations:** School of Health System Studies, Tata Institute of Social Sciences, Deonar, Mumbai, India; ESIC Medical College & PGIMSR, INDIA

## Abstract

The Baigas due to their primitive agricultural techniques, poor education status and poor population growth have been conferred the status of ‘Scheduled Tribe’ by the Government of India. The community bears the brunt of inequities, reflected in their poor nutritional and socioeconomic status. We have employed qualitative design for the study, as we wanted to understand the contextual factors for Baiga tribal children’s inferior nutrition status. Twenty in-depth interviews with the mothers of the children suffering from moderate to severe malnutrition and several other interviews were conducted with the key stakeholders like *anganwadi* workers, Integrated Child Development Scheme supervisors, Accredited Social Health Activists, public distribution system shopkeeper and registered medical practitioners. Interviews with the key informants were conducted in the Balagahat district of Madhya Pradesh. Key factors responsible for perpetuating malnutrition were then identified through thematic analysis. It was found that dissatisfaction with public services and indifferent attitude of public servants resulting in poor uptake of public services further accentuated the problem. A qualitative enquiry into the issue of high and persistent levels of malnourishment among these tribal children revealed several aspects which quantitative method may not have captured. This implies that while framing a policy for improvement in the nutrition status in such population, a holistic approach is required instead of focussing on one aspect such as supplementation of nutrition alone.

## Introduction

Malnutrition is defined as “a state in which the physical function of an individual is impaired to the point where he or she can no longer maintain adequate bodily performance process (sic) such as growth, pregnancy, lactation, physical work and resisting and recovering from disease.”[1]. Poverty is invariably linked with malnutrition, therefore, if the economic conditions of a country improves, the status of malnutrition conditions should also improve. But India doesn’t conform to the above pattern, that is, the drop in malnutrition nowhere correlates with better economic conditions in the country and thus presents a typical example of “South Asian enigma”[2]. Though, the average annual rate of stunting has shown a decline of 2.3% from 2006–14 against the rate of 1.2% in 1992–2006 (RSoC 2014)[3]. But still India does not seem to catch up with other countries with similar income levels in terms of nutritional levels of it’s children. Also at the national level, undernutrition is concentrated in a relatively small number of districts and villages, with a mere 10 percent of total villages and districts accounting for 27–28 percent of all underweight children, and a quarter of districts and villages accounting for more than half of all underweight children. More so the difference within wealth quintiles and different groups like scheduled tribe, scheduled caste and others remains distinct, as can be seen in NFHS 3 report.[4] The problem of underweight children is much severe among Schedule Tribes (54.5 of the total), Schedule Castes (47.9% of the total) and Other Backward Classes (43.2% of the total). There are 74 tribal communities designated as Scheduled Tribes, based on their low growth, low literacy, primitive levels of agriculture; the Baigas being one of them.[5] The Baigas are one of the particularly vulnerable tribal groups, spread across Chhattisgarh, Jharkhand, Bihar, Odisha, West Bengal, Madhya Pradesh and Uttar Pradesh. Poverty rates among Indian tribes is still what they were for the general population 20 years ago and same goes for their health status. A study employed the Index of Standard of Living in the Mandla district of Madhya Pradesh, found the socioeconomic status of Baigas to be poor to destitute.[6] The nutritional status of the Baiga children was worse than their rural counterparts. A study by Indian Council of Medical Research (ICMR) further confirmed the poor nutritional status amongst these primitive tribes; it found that 61 percent of the pre-school children were underweight while 24.3 percent were severely underweight; around 44.7 percent and 37 percent children were in stunting and wasting category respectively. The study also revealed the dietary pattern of Baigas: only the mean intake of cereals and calcium in micronutrients was above RDA (recommended dietary allowance), rest were below RDA.[7] A study on the socioeconomic status of households and their nutritional status has shown that children in households with poorer socioeconomic status disproportionately suffer from chronic malnutrition.[8] There is ample evidence pointing at the poor nutritional status of the children of Baiga communities, which has been discussed later in the article. But one needs to take a closer look at the factors unique to this disadvantaged section which impedes their growth and development.

## Methods

### Study site

The study was conducted in the Birsa tehsil of Balagahat, M.P. Balaghat is densely covered by forests, approximately 4775.54 sq km and the tribal population is around 17,146, which is spread among 190 villages.[9] It is endowed with natural resources like teak, sal and tendu trees.**[10]** Baigas are 22.5 percent of the total population of Balaghat, out of which around three percent are children in the age group 0–6 years (Census 2011). The respondents were from seven villages which come under a block-level ICDS (Integrated Child Development Scheme) supervisor. These villages are remotely located and quite isolated from urban cities.

### Study design and sampling

The design of the study is qualitative, it includes data from twenty in-depth interviews with the mothers of SAM (affected by severe acute malnutrition) and MAM (affected by moderate acute malnutrition) children from seven different villages to explore the social and cultural factors affecting the nutritional status among the children of Baigas. Seven formal interviews with different public servants (one ICDS supervisor, one PDS (Public Distribution System) supplier and five AWWs (anganwadi workers)) were conducted to gain insight into the stakeholders’ perspectives and the challenges they face. Both random and purposive sampling techniques were used to select the villages and the participants. In all, seven villages were selected from those pockets where Baiga population is high. Sampling of the mothers of SAM and MAM children, public servants and other stakeholders (local registered medical practitioners (RMPs), medical officer and district official) were purposive. Detailed demographic and social profiling of the villages was carried out with a specific focus on the villages under study. No a priori sample size was set for data collection; enrollment was continued until data saturation was achieved. Each of the village selected for the study had an anganwadi centre (AWC) The AWC maintains a register and keeps the record of all children enrolled at the AWC. AWCs use the anthropometric criterion of weight-for-age to identify if a child is SAM or MAM. SAM or MAM children were then identified from those registers and their health was tracked. The mothers of the affected children, who identified themselves as a Baiga were included in the sample. The interview included probing questions on social and demographic profile, means of livelihood, dietary patterns, health-seeking behavior and perceptions about the available public services.

### Data collection

The data was collected during summer (May 2015) because this is the period when there is a higher probability of finding the respondents at home, who are otherwise engaged in contractual labour in the forests. The mother/caregivers were interviewed using a pretested, semi-structured interview guide. Separate interview guides were prepared for the ICDS official, AWWs, PDS shop owner, key informant. The interviews were digitally recorded. Each interview on an average lasted up to thirty to forty minutes. All interviews were conducted and informed consent was obtained in the language participants could understand, typically Hindi, through a native-speaking interpreter if required. All interviews were transcribed from the digital recording into Hindi, then translated into English by the author. The Hindi-to-English translations were verified to ensure that they were accurate and the original responses were not lost in translation. The researcher has had no prior personal relationships with any of the participants outside the study.

### Ethics

This study was approved by the Tata Institute of Social Sciences Institutional Review Board, Mumbai. Written consent was obtained from the participants before conducting the study. Due to high rate of illiteracy among the study population, the purpose of the study and the terms for confidentiality of the data were explained to the respondents and only if the respondents understood the purpose of the study and agreed to participate, they were asked to put signature or thumb impression on the written consent form. A log of date, time and place of all interviews was kept in the field notes for documentation purposes. Following the cause of confidentiality in the informed consent, the respondents’ and their village’s names are anonymized.

### Data analysis

A qualitative study design was deployed to understand the contextual factors responsible for the poor nutritional status of the Baiga children. There are two analysis methods widely used in qualitative studies: thematic analysis and content analysis, both of which have different applications. In our study, the theoretical framework of *thematic* analysis has been used as it aids the researcher in finding meaning across the data. Also, it is a flexible method, introduced to sort the data into a structured and organized format for interpretation. Data collection was continued until saturation was achieved. Further transcribing the data requires listening to the interviews again and again and thus familiarizes the researcher with the data allowing for data immersion. The transcripts were then read to segregate the data which were then grouped according to similarities and differences. At this stage, different codes were generated. Coding categorizes the data which helps later in condensing the data further under a number of sub-themes. After generating the codes, relevant data were associated first with individual codes and then with sub-themes which were further placed under various themes. The categories and themes emerged through the narratives by inductive approach; the analysis of the narratives revealed the themes. Themes are patterns of codes that helped us to display the broader picture of what was being represented by the data.[11][12] After the themes were identified, they were illustrated with quotes, which showed different aspects of the themes. The relationship between different themes were explored and they were also seen in the light of sociocultural context within which the relationship had emerged. Analysis was done based on the thematic framework as mentioned earlier. These themes were then contrasted to make an argument in relation to the research question and the literature.

## Results

All respondents were in the age group of 20–30 years. There were 10–20 homes per village on average in the seven villages where the respondents resided. There were approximately 2–3 *tolas* (a group of about four to six homes is called ‘tolas’) in each village. A few tolas had a mixed population of different tribes. All respondents had kutcha houses made of mud, straw and the roof were made up of twigs, bamboo or baked clay, tarpaulin or plastic sheets. There was one anganwadi in each of the study village, one primary school in a study village D and a tribal hostel cum residential school in village M. Out of the 20 mothers who were interviewed, 18 had documents of identity (Table 1). The respondents were enquired if they had the basic documents like a ration card (used for buying grains, pulses from the PDS system at subsidized price), Aadhaar card (which can be used as a proof of identity throughout India). Under the Direct Benefit Transfer Scheme, the government transfers the benefit money directly to the bank account of the beneficiary, but if the beneficiary does not have any identity proof for opening a bank account, it becomes difficult for them to get any benefit from government or work in employment generation programmes like MGNREGA.

**Table 1:** Social profile of the respondents.

Village	No. of cases from each of the village
G	2
Ma	7
To	4
R	1
J	1
M	1
D	4

Out of the 20, only four respondents had both Aadhaar and ration cards; about 14 respondents had ration cards; two respondents did not have any such documents. This deprives them form availing benefits like subsidized ration from the PDS and employment under MGNREGA, which has consequences on their economic situation and hence food security.

The educational status among the respondents was very low: only four respondents had schooling up to standard 8 while two had education up to standard 5 (Table 2). The rest of respondents (14) did not have any formal education, because of which they are mostly employed as daily wage labours or contractual labours or they migrate temporarily to other places to work as labour. The poor education status and low levels of awareness of the mothers act as a barrier to improvement in the nutritional status of the child. [13] As many respondents have very low level of education, their awareness about nutritious food or health seeking behavior was comparatively low; this had an impact on the nutrition and overall health of the child as discussed in the later sections.

**Table 2:** Education level of the respondents.

Education	No. of respondents
Nil	14
Standard 1–5	2
Completed standard 8	4

### Land-holding, assets and livestock

The details about their household assets served as a proxy to asses the economic condition of the household where monthly/fixed income could not be quoted by the respondent. Of the 20, only two respondents owned land (around five acres each) while the rest did not own any land. None of the households but four owned a vehicle and a mobile phone. There was no electricity in any of the villages and the work of laying electric cables was left unfinished. Only four households had electricity (which could light only a halogen bulb) through solar panels deployed by the government. Only half of the respondents owned livestock like hen, cattle, and pigs. There was a separate barn for the cattle and pigs in a close proximity to the house. They sell this livestock at different prices: hens were sold for Rs.200–300 each, goats for Rs. 500 to Rs. 600 each while cows were sold for Rs. 2,000–4,000 each.

### Sanitation and hygiene

Out of the five As (availability, access, absorption, antibodies and allopathogens) related to nutrition, [14] absorption is affected by poor sanitation and hygiene as recurrent episodes of infection, which interfere with the absorption of nutrients and thus indirectly causing malnutrition.

#### Defecation

There were no public toilets in any of the study village, most of the villagers practise open defecation, which causes certain problems such as they have to get up early and go before sunrise; this could lead to snakebite or other physical danger. The toilets in the school in village D were non-functional and were used for storing grains. Only one household had a toilet available (open pit type latrine which was constructed with government aid under a scheme). Majority of the respondents also practised open defecation. They would go early in the morning to relieve themselves in the open and clean with leaves. This practise is common among almost all the villagers barring two who used to wash with water after relieving. Defecating in the open is the only option for the people as there were certain perceptions about usage of toilet and also, there were no community toilets in any of the study villages. Reasons stated for open defecation include the following: they were used to going in the open rather than using closed toilets which hinders their bowel movements; they have the perception that defecating in a closed area is unhygienic. Still when asked whether they would use the toilet if available a few respondents responded positively.

*We go outside*. *We wipe with leaves*. *We wash hands just like that*. *Yes*, *we would prefer going to the toilets*.

— Mrs.X16, Village G.

Washing hands before and after eating is a usual practice in all households, but only 1–2 respondents use soap to wash hands as and when it is available. Also, one respondent said that during summers, they reduce the frequency of washing hands because of the scarcity of water.

*We wash hands twice in a day*, *and in summers only once because there is scarcity of water*.

— Mrs. X13, Village-M.

But only six out of the 20 households reported washing their hands after relieving themselves, some of them using soap or ashes or soil or just plain water. When they go to relieve themselves in the morning, majority of them don’t carry water, they use leaves or stone to wipe. Washing hands after defecation was not a usual practice among many respondents.

Only two respondents said that they wash their hands multiple times and use soap sometimes only.

*We go outside*. *We wipe out with leaves*, *we wash hands just like that*. *Yes*, *we would prefer going to the toilets*.

— Mr. X19, village G

*We go to the forest*, *we just wipe ourselves using leaves*. *We wash our hands without soap; we use only water*.

— Mrs. 15, Village Ma

*Also*, *(one of the) reason for malnutrition among Baigas is that they don’t maintain cleanliness*.

— Anganwadi worker, Village Ma

#### Drinking water

The source of water for drinking as well as for other purposes in all households was bore-well water except one who had a tap at their home. They store water in big vessels and on average change the water 2–3 times a day refill only when the water gets over. Except two none of the households stated that they change or refill fresh water for drinking purpose. None of the households treated water before consumption except one who strained water before consumption.

### Dietary pattern

The diet of a majority of the households (based on their recall during last 24 hours) consisted of rice, *kodo*, *kutki*, dal (legumes, which they either produce themselves or buy from PDS) with little or no vegetables (depending on affordability). The respondents reported that meat consumption was dependent on availability of money. Some of the respondents also reported that they catch fish, crabs and prawns from the stream or the river. There was no consumption of dairy products like milk, curd, etc. in any household. The inclusion of fruits in daily diet was not reported by any household. They consume forest produce like fruits of *mahua* or *tendu* (both are fruit-bearing trees), but *mahua* fruits are usually brewed to make a local alcoholic beverage (*mand*), consumed by both Baiga men and women. *Tendu* leaves are used to roll *bidi* (by wrapping tobacco inside these *tendu* leaves (similar to cigarette). They occasionally consume green leafy vegetables like *kheda*, or *charota* from forests, which is season-dependant. It is obvious that the Baiga diet is not a balanced one; it is dictated by affordability. It comprises mostly of carbohydrates with little proteins and still less vitamins. Such diet during pregnancy may results in various kinds of deficiencies in women and can affect the healthy growth of the child.

As far as breastfeeding is concerned, most of the respondents except two started breastfeeding on the very first day of childbirth. Colostrum contains immunoglobulin IgA which provides immunity to the infant and are pivotal in protecting it against infection. The Baigas continue to breastfeed as long as the child wants to feed.

*I’ve been feeding the baby since delivery and will feed him until he wants to be suckled*.

— Mrs. X10, village Ma

*We start feeding the baby dal*, *rice*, *pasiya*, *pej at the age of 6–8 months*.

— Mrs. X16, village G

They start giving fluids like the water from the cooked lentils or rice to the child as early as 6–8 months and semi-solid food is started from the age around 7–8 months. None of the respondents prepared any specific food rich in nutrients exclusively for the child; they usually feed them whatever is cooked for the adults.

*At age of 6 months I started feeding* pasiya (rice starch), pej (cooked corn grains in fluid consistency) *boiled vegetables (to the baby)*

— Mrs. X 19, village G

All mothers we interviewed reported that they typically bought a locally prepared snack (known as *namkeen khari)* for their children. *Namkeen khari* is made from refined flour and poor quality edible oil. They were sold in clear plastic packs without any description of the brand. It mostly contains carbohydrates and hardly has any nutritional value. To keep the child engaged, so that the mothers could carry their household chores without hassles, such unhealthy snacking habit was being promoted among the children. Such snacking may satiate the child’s hunger but it will not satisfy the requirement of micronutrients or the required calorie intake of the child, leading to malnutrition.

To help in community based management of malnourished children, ICDS has set up AWCs in villages across India.[15] It is important to assess the utilization of AWC nutrition services among the respondents. AWCs are supposed to provide nutritious supplementary food, having a different menu for each day of the week, to the rural children. For instance, *dal chaval* (rice and legumes) on Monday, *lapsi* (sweet wheat porridge) on the next day, *khichadi* (concoction of legumes, rice and vegetables) on the third, and so on for each day of the week. The AWW stated that fresh food is to be prepared everyday, while supplementary food packets, some locally made nutritious food like *soya barfi*, *peanut barfi (barfi* is a type of sweet*)*, whole fruits, supplementary health mix, etc. should be given to the pregnant women as well as to the children up to three years of age. Majority of the respondents did not avail the services at AWC during pregnancy. The reason stated was that they were not aware of the services AWCs provide and no one had informed them about it. Only one-fourth of the respondents had availed services like vaccination or supplementary food at AWC during pregnancy; however, one mother said that she did not get the supplementary food given during pregnancy (on being asked about the supplementary food mentioned by the AWW)

*Yes*, *I used to go there till nine months*. *I used to get porridge*, kodai *and rice*. *No*, *I did not get vegetable*, *fruits or soya barfi or weaning food*.

— Mrs. X19, village G

Majority of the respondents sent their children to AWCs and timings of the centres are suitable for them. Four of the respondents stated that they either did not send their children to AWC at all or send them but not regularly. Reason quoted for refraining from availing the services was that the food provided at the AWC was either repetitive or of substandard quality.

(On being asked if she sends her children to AWC):

*No they don’t go there*; *the food given isn’t good*, *therefore*.

— Mrs. X3, village M

*I send them* (to AWC) *only occasionally because they sometimes don’t give food*.

— Mrs. X15, village Ma

AWCs were set up with an aim to improve nutrition among the most vulnerable children, but if the services are not being delivered properly, the mothers may not see any benefit in sending children there. It thus defies the whole purpose of setting up such centres.

### Social factors affecting nutrition

Though there are increasing efforts from the government in the recent times to improve the literacy rates among the tribes in the state by constructing residential hostels for tribes. Still, the level of literacy is quite low, which is also a reason why they are the most disadvantaged amongst the social classes. Their ancestors were living in dense forests and it was the source of food, livelihood providing for all the needs. So, for generations the tribes are used to learning life skills like hunting and gathering in open forests. The closed classroom environment for learning is very different for them and therefore has poor acceptance. This could be one among many factors along with poor infrastructure, lack of teachers and accessibility to school responsible for high drop out rate. However, this has affected them in many ways–unemployment is one of the major downside of low education. All of it has an effect on the physical as well as psychological health of the tribes in many ways–livelihood, food security, health seeking behaviour, etc.

The educational status among the respondents was very low, only four respondents finished their schooling up to standard 8 while two had education up to standard 5. The remaining 14 respondents did not have any formal education. The poor education status of women is linked to the nutritional status and it has been found to be a profound predictor in the birth of underweight babies.[13] The low levels of literacy had partly influenced their health seeking behaviour. They had firm belief in spirits, superstitions and black magic. Therefore, they rely on traditional healers or quacks who charge them exorbitantly, which has been stated as one of the reasons for debt by many respondents.

*Yes we take people for* jhaad-phoonk (the process by which the faith healer wards off evil spirits from the patient’s body)*; people cry so we get to know that the person is possessed by evil spirit*.

— Mrs. X18, village J

The Baigas approach both the quacks as well as traditional healers for minor or major episodes of illness but refrain from going to the government hospital of the village. During illnesses such as the child is weak or lactation is not enough or has stopped, they believe that some evil spirit had caused the illness/discomfort and they approach the traditional healers (*baidya*, *Baiga*, *guniya*).

*Nope*, *we call* baidya *to protect (ourselves) from the (ill effects) of evil spirits or omens*.

— Mrs. X1, village D

*We go for* jhaad-phoonk, *and we also go to the doctor*, *Dr*. *R*, *who lives 3–4 kilometres away*.

— Mrs. X3, village M

However, there was only one respondent who took the child to the public hospital when the child fell sick. She reported that there was no out of pocket expenditure incurred as the treatment was provided free of cost.

*Yes*, *the younger child fell ill; we took her to the public hospital*. *There was no expense*, *the treatment was free of cost*.

— Mrs. X7, Village Ma

As the Baigas trust the local healers more, they seek treatment from them when the children are sick. The quacks may diagnose wrongly and provide inappropriate treatment, which could cause further harm than good to the children. The underlying reason for such treatment seeking behaviour is partly lack of the knowledge about the harm the treatment may cause. Another myth that is widely believed by the Baigas, which is again the result of ignorance, is that healers using injections are better. They believe that the injections (*sujja pani* as called by the respondents) given by the quacks provide immediate relief and are better than many other forms of treatment. They are unaware which drugs are injected; for them, for any medical conditions injectable drugs are better treatment. Because of this myth, if the government doctor provide oral medicines the Baigas feel that the treatment is not good enough.

The AWWs and the health officials face challenges in convincing the mothers for the child’s immunization and girls to take iron and folic acid tablets as there is a low level of the former’s acceptance and poor knowledge about anemia or the benefits of vaccination.

*I don’t think so that they take tablet sincerely; they are scared of taking tablets*, *injections*. *Sometimes*, *if we go to a village and a group of 4–5 girls are sitting and if we tell them* (*about the benefits of taking Iron or folic acid tablets)*, *they make faces as if it is a poison*, *not a tablet*.

— ICDS Supervisor, village B

Also, the deliveries usually occur at home; only when it gets complicated, they seek assistance from the traditional healers or an RMP or other healthcare provider out there. Even though there is a low level of literacy among the respondents, majority of them stated that they initiated breastfeeding as soon as the infant is born: this refers to their cultural wisdom.

*I started breastfeeding the baby after one day of delivery*. *We start feeding semi-solid food (to the baby)*, *when it is one year old*, *we would feed dal (cooked lentils)*, *rice* …

— Mrs. X15, village Ma

*I started breastfeeding the baby as soon as he was born and yes*, *I feed him* pasiya *only now (as he is seven or eight months old)*.

— Mrs. X18, village T

Colostrum is vital for the development of immunity in the newborn. Although the duration of exclusive breastfeeding is not known, the earliest practice of feeding semi-solid food was found to start as soon as 7–8 months. However, on average semi-solid food is introduced when the baby becomes one year old. So, we can infer safely that the period of exclusive breastfeeding is maximum of one year. Also, all mothers we interviewed stated that they would continue breastfeeding the child as long as it wants to.

### Geography and climatic factors

Geography and climatic conditions influence the crops produced, food consumed and the accessibility to a region. Hilly or remote areas pose another set of challenges in the form of difficult transportation. Monsoons pose other challenges like lack of employment and poor connectivity. The roads to the villages are already in bad state; monsoons worsen the situation.

*We don’t have proper transport*, *that’s why we have to walk long distances; it’s quite tiresome*.

— Mrs. X12, village Ma

During monsoons, especially when transportation to the village (where allopathic doctor resides) is difficult, they approach quacks in the nearby villages.

*We go for* jhaad-phoonk, *also to the doctor*. . . . *Dr*. *R* (a quack), *who lives 3–4 kilometers away from our village*.

— *Mrs*. *X3*, v*illage M*

Respondents from the village on the stream (which the locals call *tada*) reported that they face acute food shortage during monsoons, because of flooding of the stream, their village is disconnected from the village where the PDS shop is located, making transportation difficult.

*During rainy season*, *there is shortage of dal*, *rice*, *because tada floods and therefore it’s not possible to go to other village to buy ration*. *We go in the forest and bring back tubers like* kareel *and* dhaunchi, *and boil it and eat it*.

— Mrs. X1, Village D

But the monsoons are also important as all Baiga households stated that they depended solely on rain for irrigation. The crops are cultivated on slopes, so the rain water runs through it and irrigates the farm. Thus the produce is largely dependent on the climate. In summers, what they call A*ashaad*, corn is sown, followed by paddy, which requires lot of water. Less rainfall means poorer yield which in turn affects the food availability.

*Yes*, *when we fall ill*, *or when there is a shortage of food during summers*, *we borrow 1000 or 1500 rupees from fellow villagers*. *Every year we borrow 1000–3000 rupees*.

— Mrs. X14, Village Ma

These regions are usually left behind in terms of development. For instance, there is a lack of employment opportunities, which hit them hard, especially during monsoons. Even getting a daily wages work is difficult, resulting in shortage of money to buy food, hence affecting the nutrition adversely.

*In monsoons*, *there is lack of work or daily labour*. *So*, *there is scarcity of food*. *We find work somewhere like filling the tractors with soil*, *etc*., *else we take out loan*.

— Mrs. X8, Village Ma

### Economic factors

Due to poor literacy rate and poor levels of awareness, the Baigas are unable to find a stable employment and are exploited as labours, which fetches them meagre income. This is also because of their low negotiation capacity as they are perceived as ignorant and are poor. The findings of the study present a grim picture of the economic status of the tribe, where none of the respondents had any stable source of income. Because of low employment, almost all respondents were in caught the trap of debt. Therefore, it is highly unlikely that the households were buying and consuming food of required quality or quantity. As poverty restricts the number and the quality of their meals as well as cooking methods.[16]. The decision on buying food items in poor households is dictated more by affordability than by nutritional value, which affects the nutrition status of the child. The excerpts below reveal their poor socioeconomic status and its influence on their food.

*At home we cook fish or a jackfruit*. *We catch fish from the pond; that is the only place we can catch them*. *If money was there*, *then we would buy fish*, *but we don’t have money so how can we buy and eat (fish)*.

— Mrs.X7, Village T

This excerpt explains the inability of the household to buy food due to poor financial conditions. Although they would like to consume meat more often, since they cannot afford to buy, the tribes go to the nearby streams and fresh water bodies to catch fish occasionally. Food items which they cannot usually afford must be bought in small quantities. And because of the lesser quantity, the children’s share may be too scant to meet the appropriate dietary requirements.

*We start feeding the child when s/he is 7–8 months old; what else can I feed the child*, *madam*, *only dal*, *rice*, *vegetables (*that I cook for everyone).

— Mrs. 19, Village G

The absence of a stable source of income leaves them in abject poverty, to the extent that they could barely afford two square meals, let alone complete nutrition. There is a big question mark on food security among all households as they live hand-to–mouth. If they cannot find any job or there is a sudden expenditure due to some medical condition, it poses a challenge for their food security.

*Yes*, *we cook roti and dal but if there is nothing then we can hardly do (cook) anything*; *it becomes problematic*.

— Mrs. X2, Village T

#### Means of livelihood

The root cause of poverty is a lack of employment opportunities discussed earlier. Due to lack of government employment schemes and delayed and/or underpaid work under MGNREGA, daily wages job is for a majority households, the most frequent source of income; these jobs are the most common economic activity among Baiga households. Another reason for food insecurity is the unpredictable agricultural output. Although most of the households practise farming, agricultural produce seldom exceeds the requirement for consumption at home. This is partly due to old methods of farming, which the tribes practise. Rainwater is still the mainstay for irrigation and cultivation is on slopes, so that the rainwater runs off and irrigates the crops. Shortage in rainfall is, therefore, bound to affect the produce, thus posing a challenge to their food security, as the household storage of grain may run short, well before the next season.

*(Shortage of food occurs) in* Kuwar *(April–May) and* Jaith *(September–October)*. *(During)* navratra, *shortage of rice occurs as a shortage of money and we go place to place (in search of work) if any daily wages work is available*, *otherwise we starve and suffer*.

— Mrs. X7, village T

For income, the members depend on varied means of livelihood during different times of the year. The nature of employment is seasonal. Most of the households work as daily wage workers or they work in the forest as contractual labour during the year as and when called by the contractors. They collect and sell *mahua* fruits, *tendu* leaves and other forest produce like *harra*, *charota*, *kheda*, *chaar*, honey, tubers, fish, etc. This generates an average income of Rs. 1000 and occasionally up to Rs. 5000 every year. Most of the respondents stated bamboo cutting as the highest income generating activity, which lasts for two to three months (from September to November) every year and depends on how much bamboo has been cut. A few of them stated that they collect forest produce on their own and sell whatever little they could spare to earn some cash. Farming is another means of livelihood but the yield is sold only when there is a surplus. Minimum annual earning reported from farming varies from Rs. 400 to Rs. 500 and a maximum of Rs. 8400 per year. During seasons when there is no source of earning from forest produce or agriculture, the tribals have to search for daily wages. Some migrate to other states and take up labor work for two to three months. The reported income they receive was as low as Rs 4000 and up to Rs. 16,000 during their migration period. Some other sources of income include selling livestock and MGNREGA. Under MNREGA, only those who have a bank account could work as daily wages worker. Many respondents did not have one. Only four respondents had worked under MGNREGA and some of them did not receive their wages.

*No*, *where do we get it (the wage after working under MGNREGA) regularly* …

— Mrs. X12, Village Ma

By this estimation, hardly any of the Baiga earns up to Rs. two lakh per year. Earlier, the Baigas would go more frequently to the forests than in recent times since they used to rely completely on the forest for their livelihood. However, the imposition of new laws restricting their access to forests, and apathy of the administration towards these forest-dwellers has left them without many options but to search for menial jobs every other day to make their ends meet.

#### Household expenditure

The average income of Baiga households based on reported earnings is between Rs.2000 to Rs. 5000 per month. This may vary during different months because in some seasons as mentioned before occupations like bamboo cutting, agricultural produce may yield more income. The average consolidated expenditure on food by the households was around Rs. 3000 to Rs. 4000 per month, including expenditure on vegetables, spices, grains, meat and miscellaneous items for children (*khari* and *biscuit*), which cost approximately Rs. 10 to Rs. 20 per day.

*We buy (food) only in little amounts*. *We can buy (food*, *clothes*, *etc*.*) only if we have money*. *Out of the earnings about 500–600 is spent in a week*. *If there is no money*, *what can we do*!*’*

— Mrs. X7, Village T

Expenditure on items other than food was around Rs. 3000–Rs. 5000 per month on average, including transportation, clothing and miscellaneous. Other than this, the occasional and unforeseen expenditures could be Rs 4,000–Rs. 5,000 every year such as on rituals and festivals like *Diwali*, *Holi*, *Hariyali*, *Chatthi*, and *Barsa*. Majority of the households spend a lot on *mand* (locally brewed alcohol) during these occasions.

#### Loans

All participants had borrowed cash or kind some time or other. The most common reasons stated for borrowing were medical expenses and shortage of food; others were weddings and other social functions like *Barsa* and *Chatthi* (both are events related to birth of a child), *anna prashan* (when the baby is fed semi-solid food for the first time), *godbharai* (baby shower), *Diwali*, *navakhai*, Holi, *Phagun*.

*We celebrate* Barsa, Chatthi. *We cook dal and rice*. *Lot of money is spent*, *around Rs*. *1000 on items like* kodo (a variety of millet), *green leafy vegetable*, mand, mahua, *etc*.

— Mrs. X11, village D

*Yes*, Chatthi *and* Barsa *are celebrated and around Rs*. *20*,*000 are spent on the occasions*. *There is a feast*. *We buy new cloths*. *Arrange sound system*, *etc*. *and call our relatives*.

— Mrs. X1, village D

Most of the respondents stated that because of lack of daily wage jobs, especially during rainy seasons when the stream would overflow and make the access to the PDS shop difficult, the households face food scarcity. They have little money as well as they have no other choice but to borrow rice or other grains from fellow villagers for their everyday consumption; they usually pay the debt back in kind. Any episode of illness, however minor, could worsen their financial condition. The respondents approach faith healers or quacks for health emergencies or any illness instead of public health facility, which costs them dearly, from Rs. 200–Rs. 300 to Rs. 2,000–Rs. 3,000 per visit. Only a few respondents had separate savings for medical emergencies.

### Cultural and traditional practices affecting nutrition

#### Primitive farming practices

“Baigas still practise their traditional method of cultivation, though many of them have been persuaded to switch to and use a plough. Although many of the Baigas plough the land for cultivation, they do so with a lot of regret. *Bewar* (slash and burn type of farming) is their tradition; it is in their blood; it is the mark of their race; it is the sole reminder that they were once the kings of the forest. God himself came to their great ancestors as they were eating roots in the jungle, and showed them how to do *bewar*, giving them the first seeds from his own hands. The Baigas are passionate about *bewar*. I have no doubt that this almost religious devotion is connected to their reverence for Mother Earth. Now because of starving situations and the current strange laws, they have to dishonor her, lacerating her fair breasts with the plough.”

This is an excerpt from the book ‘Leaves from the Jungle’ (1936) by Verrier Elwin, a British-born anthropologist who worked extensively on tribes of Central India–*Baigas*, *Gonds*, *Madhiya and Muria*.[17][18]. *Bewar*, which the Baigas have been practising since ages is based on their religious belief that god has shown them how to sow seeds in the ashes of burnt trees. It has become a part of their culture and identity. During British colonisation, the administration interfered with the Baigas’ way of living, as their interests were in contrast with many of the practices of Baigas: *bewar* was one of them. *Bewar* was seen as a detrimental practice which if not stopped would lead to increased deforestation. The Baigas under the colonial rule were thus forced to stop practising *bewar* and they had no other choice but start ploughing the land for cultivation.

#### Attitude towards health and treatment seeking behavior

The attitudes towards health seeking behaviour of the Baigas is largely shaped by their culture and traditional beliefs. As mentioned earlier, these tribes were forest dwellers, and therefore they had great knowledge of medicinal plants and herbs used for various medical conditions. Also, there perception of an illness could differ from the most prevalent notions. For instance, they believe that when some person is ill, he/she is possessed by some bad spirits, and therefore they still believe in faith healers for the treatment of many illnesses, though this perception is slowly changing. The cultural beliefs affect the treatment seeking behavior of the Baigas. They believe that evil spirits are responsible for the episodes of illnesses. So, when the child is weak or lactation is problematic, they tend to approach traditional healers (known as *baidya*, *Baiga*, *guniya*).

*Yes*, *we go for* jhaad-phoonk *when the child is ill; also if we have problems concerning breastmilk*.

— Mrs. X4, Village T

Rs. 1000–Rs. 1500 are charged every time they approach the faith healers for *jhaad-phoonk* they are paid in kind (sacrifice hen, *mand a kind of alcohol*), which again costs them around 800/- to 1,000/- some respondents even spent as high as 20,000/-.

No (we don’t approach any qualified Dr.) we call ‘*baidya’* to protect from evil spirits, omen.

— Mrs. 11, Village D

Another instance is their perception towards pregnancy. They do not acknowledge pregnancy as a condition that requires more nutrition and care, which is obviously a false belief. This perception is likely to impact the health if pregnant women and development of fetus. All the women stated that during pregnancy they used to perform all household work like cooking, cleaning, fetching water etc. Also going for daily wage work to earn a living during pregnancy shows that the women have to engage in labour work because of poverty and food insecurity, and this can be derived from their narratives.

*Yes I used to do all the household work and used to go to earn (daily labor) too*.

—Mrs. X6, village T

Lack of awareness regarding extra nutritional requirements and other precautions during pregnancy is quite evident in the tribe. Be it refraining from physical exertion or taking nutritious food or dietary supplements. They do not seem to have the knowledge about the requirement of additional calories and vitamins during pregnancy, which may result in inadequate food intake and poor nutrition status affecting the growth and development of the foetus. None of the respondents reported any change in the frequency and quantity of food intake or in the dietary pattern or inclusion of fruits, milk, etc. in the meals or otherwise before and after pregnancy. Pregnant women eat the same food cooked for everyone else at home.

*No*, *I ate the usual food* (dal, rice, some vegetables) *and in the same quantity as I used to eat before (my pregnancy)*.

—Mrs. X17, Village R

The Baigas undoubtedly have a rich culture and traditional wisdom of their own, which has helped them survive in the dense forests in harmony with the nature since ages. But some of the superstitions or beliefs are affecting their health and development, leaving them in an impoverished state.

### Dissatisfaction with the public services

The tribe traditionally used to have chieftains or some elderly person who would look after and resolve the issues of the community[18]. The abrupt shift from their traditional institutions to the modern ones have not been taken up by the tribe positively and for obvious reasons. [18] There is a meager participation and utilization of public institutions such as panchayat among the tribes, which is a constitutional body consisting of elected representatives of the community. The idea behind the panchayat system is to decentralise the governance and delegate powers to the elected representatives called Panchayati Raj Institutions (PRIs) empowered to function as institutions of self government and to prepare plans for economic development, social justice and their empowerment. PRI constitutes the bedrock for the implementation of most of rural development programmes.**[19]** It has been described by an author as just a formal institution with no role in various tasks. Most of the respondents are not satisfied with the functioning of AWCs and the work of the panchayat since they have failed to provide them even the basic amenities like roads, electricity and employment through MNREGA.

The attendance on an average in the AWCs is low. Not all children who are enrolled go to the AWC everyday. The reason stated by the respondents for not sending the children to AWC was that the food served at the centre was repetitive and unpalatable despite there is a different menu for each day of the week.

*My child doesn’t go anymore (to* anganwadi*)*. *Because their menu is repetitive*. *I can provide him better at home*.

—Mrs. X17, village R

The officials also highlighted the issue of availability of vegetables for meals. AWWs can buy them only from the *haat* (local market), which is usually open once in ten days or so. The transportation of vegetables is difficult and the AWWs now grow local vegetables like pumpkin, bottle gourd, etc. in the backyard and use them for meals. The AWC is supposed to be tracking malnourished children but the AWWs were hardly aware of the different criteria for identifying a malnourished child. For instance, a month ago, a child was considered ‘underweight’ or ‘severely underweight’ or severely malnourished and the next month he was not, meaning his weight had increased and he was no longer underweight. The chances of which are highly unlikely.

The public health services uptake among the tribes is negligible, the reason may be varied. For instance many respondents weren’t aware that the treatment provided at the government hospital is free of cost. Majority of the respondents, around 14 of them never went to the public hospital for any treatment and there was no specific reason stated, while some of them who never went to the public hospital did so for reasons- like long distance from their village, belief in the quacks and faith healers more than the doctors at the public hospital.

*It’s (government hospital) in village B*; *it’s about 35 km away*. *No*, *we never go (there) because it is quite far (from here)*.

—Mrs. X1, village D

Only five respondents had availed the services of the government hospital for delivery or treatment for their child’s illness. Also, the Baigas, as described earlier, are superstitious and it has implications for their treatment seeking behavior. Another reason is the easy access to faith healers or quacks who are usually set shop close to the village and also visit their homes, if required. The confidence that the informal health provider will be available any time is what makes them trust these providers strongly and because of this they may not be approaching the government hospitals.

*Why the Baigas don’t approach public hospitals is because the medicines given (there) are of sub-standard quality*; *they don’t believe in treatment in the government hospitals; another reason is that the Baigas believe that injections could cure diseases*, *which are seldom given in the public settings*.

— A key informant, Village D

There is an attitude of ‘victim blaming’ among the public servants, as the tribal are seen as ignorant forest dwellers and uneducated people. According to the excerpts from what they had to say, the tribes should be held responsible for their own poor health status, which, as we show below from the data we have collected, is not true.

*These people are habituated to eat tubers and forest produce (*kand-bhus*)*, *(as a result) their children should be much healthier than our children*.

— AWW, village T

This attitude may be responsible for the lack of trust between the tribe and the officials. This may have implications in a way, because the tribe may refrain from availing public services.

*It is due to the negligence of parents; now*, *see*, *the children are theirs and therefore they have to take care of them; anganwadi workers can take care* (of their children) *only for 4–5 hours (a day)*. *They (parents) go to the forest*, *sometimes leaving their child back at home*. *You might have observed sometimes that they carry their children on their back*, *tied to it with a cloth*, *even if the child is eight days old*.

— ICDS Supervisor

According to the fourth round of National Family Health Survey (2015–16) in Balaghat, only 23.8% of the children in the age group of 12–23 months were immunized while 76.2% were partially immunized and the rest are not immunized at all.[20] Though the government provides vaccination free of cost to pregnant women, infants and children, none of the respondents were aware of how many vaccines the child had received or about the schedule of vaccination. This shows that there is a need for improving their awareness about the advantages of complete vaccination. Because a malnourished child is prone to many diseases and vaccination will boost its immunity. It is also important to prevent further medical complications.

While some of them were reluctant to get their child vaccinated, there are some misconceptions and fear due to which the health providers find it difficult to convince them for vaccination.

*(Once) I saw that one of the child’s arm had swollen and he was in a lot of pain after they vaccinated him*. *Therefore*, *I don’t want (to vaccinate my child)* … *they leave us after vaccinating the child*. *What if the child falls sick after they leave*? *Whom will I approach*?

— Mrs.X6, village T

## Discussion

Historically, tribal communities are characterised by a lifestyle distinct from the agrarian communities. They subsist on different combinations of shifting cultivation and hunting and gathering of forest products: all activities closely linked with forests. We need to realize that we have disrupted an already self-sufficient, orderly society which had evolved in harmony with the nature to such an extent that going back is not possible anymore and catching up with the present is a struggle. Various acts and laws enacted for the betterment of the tribal populations have caused more harm than good. The Indian Forest Act 1927 categorizes forests into reserve forests, protected forests and village forests, out of which village communities could access only the village forests (Indian Forest Act 1927, Ch. II, Section 110, Ch. III. IV, Section 111). Because the tribal populations have been inhabiting the dense forests and consider themselves the guardians of the forests rather than an owner, alienating them from the forests has affected their lives adversely. The tribes in India have been struggling since the era of British colonisation. As the British wanted to ‘civilize’ them and therefore they curbed and tried to abolish many of their age old practices. For instance, the Baigas have been practising slash and burn type of farming since ages but this was considered by the British as detrimental to the forests. So, they restricted their access to the forests and also pushed them to stop the practice of *bewar* and instead cultivate in restricted piece of land. Since then the tribes have been denounced and displaced from their own environment to create wildlife parks and sanctuaries.[21] There has been many non-tribal private purchases of land by the state, proclaiming that is being done in the public interest. The answer to whether the tribes were consulted for these purchases is also ambiguous, the amount of consultation for such purchases would have been minimal. [22] The laws may act as a tool at times by which the government may justify acquisition of tribal land for public welfare; one such law is the Land Acquisition Act of 1894. [22] Despite the provision of special constitutional and legal rights, the tribal population is one of the most displaced, vulnerable and poorest of all sections of the society.[23] These events have been affecting the tribes since generations and has wrecked the economical, cultural and social aspects of their life.

A consequence of the constant deprivation of the tribe’s rights and dignity is that today the tribal belts, which overlap with the country’s major forest areas, are also areas with the highest concentrations of poverty. Also, it has been found that though malnutrition rates have reduced across all social classes during several past years, disparities still exist in household wealth and female education[24][25] Receiving education is the only way to break the vicious cycle of poverty as it will provide them better opportunities in getting stable jobs and in turn improving their economic condition. Also, the poor health status can be partly attributed to the lack of education and awareness about their own rights and well-being. Majority of the households under study were in the trap of poverty due to lack of employment opportunities. A study on 400 Baiga respondents found that only 31.75 per cent of them were educated and girls were at a higher disadvantage.[26] The educated households were earning more and had lesser debts than the uneducated respondents.[26] This shows that education directly impacts the economic conditions by improving awareness about the public schemes and influencing their health seeking behaviours. Illiteracy along with their cultural beliefs affects their treatment seeking behaviour too. As majority of the households still believe in ghosts, spirits and seek treatment from the informal providers and faith healers.

Sanitary practices among Baigas is poor owing to reasons such as lack of awareness about the ill effects of open defecation unhygienic practices, water shortage during summers, lack of community toilets, etc.[27] It is a well-established fact that poor sanitary conditions are highly correlated to malnourishment. An analysis of a pooled data of 1393 children showed that the probability of stunting increases by 2.5 per cent per an episode of diarrhoea and by 24 months of age, 25 per cent of stunting was attributable to five or more episodes of diarrhoea.[28] The recurrent bouts of infection impedes the normal growth and development of the children. So, there is a pressing need to create awareness about the importance of hygienic practices by facilitating them with infrastructure to maintain hygiene and proper sanitary conditions. For instance, a study suggests that a mean reduction of 37.5 per cent in diseases which spread through feco-oral conditions is possible in developing countries following the introduction of water supply, sanitation.[29]

The geographical and climatic conditions also pose challenges in terms of cultivation or difficulty in inter-transportation between neighboring villages during monsoons, due to which access to a PDS becomes difficult. Food availability during certain periods of the year such as summers and monsoons is problematic. Many respondents stated that there was a shortage of food and they had to borrow food. In such situations, it is highly unlikely that a person will be able to get a balanced diet in adequate quantity and of acceptable quality. The health and nutrition survey carried out by ICMR (1988–89) on different tribes of Madhya Pradesh found that mean cereal intake among the tribes was 425 ±11.6 gm/cu/day; consumption of pulses was much less, 17.6 ± 2.8 gm/cu/day as compared to RDA (40 gm). Oil and fat consumption was negligible (2.2 ± 0.7 ml/cu/day). The mean energy intake was 1615 ± 57.2 kcal/day with protein intake of 50.2 ± 1.9 gm/day.[30] The findings of the current study too corroborate it. A major portion of Baiga diet consists of carbohydrates, with very little vegetables, fruits, no milk or milk products and it seldom includes meat, fish or any other sources of protein.

Another emergent theme is the dissatisfaction with public services as no basic amenities like roads, electricity, drinking water are provided to them yet. Also, the respondents were reluctant to send their children to AWCs because of the unsatisfactory food being served, which defies the purpose of establishing them. If these public services are not available then the nutrition status of the children will hardly improve. If the child falls ill or is weak, they seek treatment from faith healers or quacks rather than from public hospitals. This implies that it is necessary that the state takes cognizance of their needs and takes steps towards making public services more reliable and preferable.

## Conclusion

As for the Baigas, the introduction of so-called ‘developmental policies’ of the government disrupted their self-reliant traditional ways of life and left them at the vagaries of the state. Their culture celebrated and fostered their close bond with the nature at the same time also emphasizing communal ownership and communal consumption, closely-knit kinship structures and minimal hierarchies. A deep-seated perception in India is that tribes are primitive communities with little or no order in society, and the developmental policies are therefore directed towards making them more civilized and socio-culturally evolved. Obviously, such a view is a product of the dominant culture’s prejudice against, and ignorance of, the culture of both settled and nomadic tribal peoples, particularly those deemed primitives, since each of these groups, of course, used to have its own customs, traditions and laws.

Apart from the administration’s indifferent attitude, the poor education status strengthens their beliefs in superstitions, which acts a barrier for the administration to convince them to take up health services or provide them health education. The poor nutritional status of Baiga children could be attributed broadly to poor socioeconomic status of the Baiga households–lack of employment opportunities, poor education status, strong cultural and religious beliefs, geographic and climatic conditions, which affect the quality, quantity as well as availability of food, and treatment seeking behavior impacting the child’s nutrition status.

The administration should emphasize the detriments of open defecation and promote awareness regarding hygienic practices as well as how it can improve their conditions. Facilitation of aids for construction of personal or community toilets may encourage them to use it. Generating employment opportunities, which makes use of their traditional skills like collecting honey, herbs, etc. from forests, could prove helpful in addressing the problem of unemployment. As their poor economic condition is the root cause affecting the food availability, the Baiga women go to search for daily labour work even during pregnancy to earn some cash.

Lack of even basic amenities adversely affects the children’s nutrition status directly or indirectly. There is a need to sensitize the public servants serving in these tribal areas so that they have a better understanding of the tribes. To improve the service uptake of public services, it is necessary to remove some bottlenecks such as illiteracy, geographical barriers and sensitivity towards the tribes. The onus lies on the state and its administration to facilitate services in a way that encourages the tribal people to access public institutions as well as to utilize them. Until, the basic services and needs of the tribes are satisfied, just providing supplementary nutrition is hardly going to bring any change in the children’s nutrition status and overall growth and development. The policies schemes designed for them must be framed by including their inputs instead of using the top-down approach. For instance, a toilet which was built adjacent to the primary school was utilized for storing grains and not for the original purpose. Including them and encouraging their participation will give them a sense of ownership and will help bridge the gap of trust between both parties. Thus, the consistently high prevalence of malnutrition among these children cannot be attributed to a single factor; actually it is a result of the cascading effect of a number of factors which are interlinked among themselves. For policymakers it is therefore important to frame policies which address the issue in a holistic way, because targeting a single factor will not be enough.

## Supporting information

S1 File S1_Language editing track changes. File with language editing.
(PDF)

S2 File S2_Interview guide (English). Interview guide in English.
(DOCX)

S3 File S3_Interview guide (Hindi). Interview guide in Hindi.
(PDF)
