# Supplementary material for: Socioeconomic determinants of nutritional status among ‘Baiga’ tribal children In Balaghat district of Madhya Pradesh: A qualitative study
Source: PLoS One. 2019 Nov 21;14(11):e0225119. doi: 10.1371/journal.pone.0225119 (PMC6874081; doi:10.1371/journal.pone.0225119)
Supplement: S2 File — Interview guide in English. (DOCX) [file pone.0225119.s002.docx]

INTERVIEW GUIDE

Respondent Name:

Address: Age education

Religion: Caste:

Type of family: nuclear/joint family Basic documents: BPL card/ ration card/Others, Specify

| Name | Relationship with respondent | Age/Sex | Education | Marital status | Income | Occupation & remarks |
| --- | --- | --- | --- | --- | --- | --- |
|  |  |  |  |  |  |  |
|  |  |  |  |  |  |  |

These questions will help us know the sources of livelihood, resources, assets and hence the level of poverty of the household

| These questions will help us know the sources of livelihood, resources, assets and hence the level of poverty of the household | | | | |
| --- | --- | --- | --- | --- |
| Livelihood resoubfdfkjbrces  Livelihood resources | Products used at home  Products used at home | | If sold then income generated  Season/yearly | |
| Agricultural land -Does your family has any land holding, If yes then(cultivable/non-cultivable), how many acres of land are you holding, how do you irrigate it? Which crops do you cultivate? Do you sell the crop or stock it? |  | |  | |
| Livestock (no. and types of livestock) |  | |  | |
| Forest products (frequency of visit to the forest, number and name of products that are collected) |  | |  | |
| Assets:  (For e.g. kuccha/pucca house electricity, bike, fan etc.) | | | | |
| In this section we discuss about the migrational status of the family, how frequently they migrate and how many members migrate for earning livelihood. | | | |  |
| Please describe the Migration status:  -Did anyone from your family migrated for earning? How many of you migrate? Which period of the year do you usually migrate?    -Which employment gives you bulk of income for your household? | |  | |  |
| The living conditions and lifestyle of the household is reflected by the following questions, and it implies how they are linked to it. | | | |  |
| I would like to know the daily living activity and condition of the family:  -How is the structure of the house? (Probe on type of house, room in house, open space, livestock shed).  -Where is the Source of drinking water supply, how do you store it? How often do you change it? Do you treat the water before drinking?  -What type of cooking fuel used in the house?  -How do you maintain cleanliness in your house?  -How is the waste disposal done, what type of sewage system do you have?  -Do you have toilet facilities at home, which type of toilet are you having, what are your sanitary practices? Where do you take bath?  -How many times in a day you wash your hands? | |  | |  |
| I would like to know the availability of work/ job for the family in the village.  Does any family member needs to migrate in search of work? If yes what kind of migration, then  If agricultural land, then  If livestock’s, then  If forest collection, then  House work | | Which are the employment where highest number of days households work?  Does any migration for employment from a house at any time in a year?  Who migrate and where?  What type of employment they access there?  For how many days they are employed  What type of food cultivated in a farm?  How is the irrigation facility available in a farm?  How many member of the family employed in a farm and for how many days?  How many members involved in care of the livestock in a household?  How you manage the livestock?  How many members involved in a forest work?  Which season is the most suitable for forest collection  Who all are involved in a house work?  How much time it will take daily for house work? | |  |
| The questions in this section reveal the dietary habits and practices, as the nutrition that the child and mother gets during the developing years and afterwards is an important aspect of malnutrition. | | | |  |
| I would like to know the dietary practices of the family in a year.  - What all did you had yesterday (since morning to evening)? (Desc. frequency, Quantity of the food)  - Any special diet on specific occasions? What do you usually give in meals to the children (frequency & expenditure)?  - Is the same food served for all members, or something else to the child?  -Describe your cooking practices (washing vegetables before use, freshly prepare meals every time, etc.)  - How is the storage system for food items in a house?  -Which time in the year is it difficult to procure food items? Why? How do you cope up with it?  -Any changes in diet, when the child falls what all do you give? For how many days do you give him special diet?  -At which did the child got diagnosed with malnutrition? | |  | |  |
| In this section we are going to discuss the expenditure and break down of the expenditure on various food and non-food items, medical expenditure. | | | |  |
| What are the different expenses in a year of the household?  -  - Daily/weekly expenditure on food items.  -How frequently do you buy ration? What all does it includes? what amount do you spend on ration monthly/quarterly?  - List the expenditure on non-food items (frequency of buying clothes & expenditure, education, travelling, ocassional spending on functions).  -Did you ever take loan from any source to compensate expenditure? Specify the purpose, source.  - Are you having any loan, specify the source, amount and purpose?  -Any special food expenses made for children?  -Are you having any savings for emergency health condition?  Expenditure on illness | | \| Food expenditure \|  \| \| --- \| --- \| \| Non food expenditure \|  \|  \| Minor illness(in last 15 days) \| Expenditure \| \| --- \| --- \| \|  \|  \| \| Major illness(in last1ear) \| Expenditure \| | |  |
| The cultural practices are relevant since they are linked to mother and child care as well as dietary practices | | | |  |
| Describe your cultural practices related to maternal and child health care?  (maternal care, breast feeding and weaning food practices and health care utilization during illness.)  -What are the rituals associated with pregnancy and birth of new child?  -When did you get married? At which age did you conceive for the first time?  -Did you suffered with any illness during pregnancy?  -You had institutional delivery or home delivery (yes/no), why?  -Did you get yourself registered in the hospital ? Did you had Antenatal checkups? If yes, then how many and where?  -What are the breast feeding practices, till what age does it continues?When did you start breast feeding?  - Did you breast feed your child when you are ill?  -At which age did you start feeding semi- solid food items other than mother’s milk to your child? Please list those items.  -What is the interval between each pregnancy?  -If the new born/child falls ill, do you take them to traditional/faith healers or doctors, which one do you prefer and why? If yes  Where you avail treatment  -Where do people in your village avail the maternity and child health related services normally?  -What kind and quantity of diet is given to pregnant women and mother of newborn?  -Do you feel your child is weak as compared to other children of same age group or falls ill frequently?  -What are the daily chores in you were engaged in when you were pregnant, till which month?    -Who feed the children during your absence from house?  -Is there any particular belief associated with birth, feeding and weaning practices and illnesses? | |  | |  |
| These questions will highlight the perceptions of respondents about working of public institutions and its services. | | | |  |
| What are the various public services available in a village and its functioning?  --What all health facilities are available in your village? Is the doctor available all the time? What type of maternal care services are available in your village?  -Is there any door to door health facility available in your village? Is there any faith healer in the village  -Does your village have a school? Are the meals given regularly in the mid day meal scheme? Is the teacher coming regularly and child attending the school regularly?    -Is there any Anganwadi in your village, does it remain open on all days?  -Is your child enrolled in Anganwadi? Is your child attending it regularly?  -What are the timings of anganwadi center? Are the timings feasible to you?  -What type of food served in Anganwadi? Do you think it is sufficient and nutritious?  -How far is the PDS system from your village?  -What type of food items provided under the PDS? Is the ration given regularly?  - Is there availability of road and transport facility available till nearby Taluka place?  -Does government provide any employment opportunity under MNREGA? How many members of your family are engaged and what are the wages, for how many days?  Where people avail the maternity and child health related services in a village? why  Is there any door step health facility available in a village mostly during rainy season?  Do ANM/ ASHA visit your HH during pregnancy and make you aware about pregnancy related information (importance of monthly checkup, pregnancy related complication, importance of nutrition, breast feeding and weaning food).  -are the water, sanitation, electricity, road services provided through Gram Panchayat ? Are they satisfactory?  I would like to know the cultural practices of the family. Various rituals performed in a community at a various occasion.  What are the ritual practices in a house?  What are the beliefs associated with daily living?  Who is the decision maker in the house | |  | |  |

Key informant interview

Questions to Anganwadi workers:

Name: Age/sex:

Residential address: Year of service:

Institution of working:

Number of students enrolled: Average number of students regularly attending:

Number of SAM children: Number of MAM children:

Which programmes are running in your centre currently? are there any programme exclusively for mother and child?

Are the children and pregnant women regularly attending the anganwadi?

What kind of diet is provided to the children in anganwadi. What is the quantity and frequency?

Are the children eating the food at the centre or you give them to take it back home?

Is pregnant women provided food in center? If yes, then what food items are provided and in what quantity?

What precaution do you maintain to ensure hygiene and quality of food?

Do you know about malnutrition? Are there any programmes running in your centre to address it?

What are the challenges that you face while providing services under Anganwadi?

What difficulties do you incur while availing food, funds and salary of employees in Anganwadi .

According to you what are the reasons for prevalence of malnutrition among children according to you (any beliefs or practices in particular…).and what ways do you suggest to tackle it?

**Questions to PDS supplier:**

Name: Age/sex

Address: shop since how many years:

Institution on working:

Government norms of distribution:

Which food items are distributed by your ration shop? At what interval is the ration being distributed, is the supply regular?

Do you think prices are affordable and quantity is enough for them?

Are there any hurdles they are facing in procuring the food items, if yes please explain.

Do you incur any problem while procuring food items for the shop? Please explain.

What is the distance of ration shop from nearby villages? How many villages it covers?

How far is the distance from the farthest village?

What food item you suggest should include in list.

Question to NGO

Head of the Ngo: Age/Sex:

Address: year of establishment:

Institution of working: Number of year since working:

What is the scenario of malnutrition in your area? What do you think are the reasons for it?

Which are the different types of illness children suffer under the age of 5 in the area?

How is the health services utilization pattern among tribes in area related to mother and child illness?

Did you feel public services play an important role in malnutrition and how?

What are the services provided through NGO to address malnutrition?

What can be done according to you to reduced malnutrition in your area?

Questions to ANM/ASHA

Name: Age/Sex

Addresss: year of service:

Institution of working:

What services are provided to pregnant mothers and children under age 5?

What are the common illnesses they suffer?

How is the utilization of the health services?

What difficulties do you incur while providing services to the community?

According to you why there is malnutrition in community?

Where health system can help to reduce the incidence of malnutrition.
