# Supplementary material for: Socioeconomic determinants of nutritional status among ‘Baiga’ tribal children In Balaghat district of Madhya Pradesh: A qualitative study
Source: PLoS One. 2019 Nov 21;14(11):e0225119. doi: 10.1371/journal.pone.0225119 (PMC6874081; doi:10.1371/journal.pone.0225119)
Supplement: S3 File — Interview guide in Hindi. (PDF) [file pone.0225119.s003.pdf]

संलग्नक (ANNEXURE) 2:

साक्षात्कार पथप्रदर्शक :—

उत्तर देने वाले का नाम :—

पता :— उम्र :— शिक्षा :—

धर्म :— जाति :—

परिवार का प्रकार :— एकल / संयुक्त परिवार मूल दस्तावेज :— बीपीएल कार्ड/राशन कार्ड/अन्य

नाम	उत्तर देने वाले के साथ संबंध	उम्र / लिंग	शिक्षा	वैवाहिक स्थिति	आय	व्यवसाय तथा टिप्पणी

ये प्रश्न हमें आजीविका के स्रोत, संसाधन, संपत्ति और गृहस्थी की गरीबी के स्तर को जानने में मदद करेंगे।		
आजीविका के संसाधन	गृह के उपयोग में आने वाले उत्पाद	अगर बेचा गया तो कितनी आय ऋतु/वार्षिक

कृषि भूमि–आपके परिवार के पास कोई भी भूमि है– यदि हाँ तो (खेती योग्य/गैर खेती योग्य) तो आपके पास कितनी एकड़ भूमि है? आपके पास सिचाई की क्या सुविधा है? आप किन फसलों की खेती करते है? आप फसल बेचतें है या परिवार के उपयोग लिये रखते है?		
पशुधन (संख्या एवं प्रकार)		
वन उत्पाद (एकत्र किए जाने वाले उत्पादों की संख्या और वन भ्रमण की आवृत्ति)		
संपत्ति (कच्चा/पक्का मकान, बिजली की व्यवस्था, मोटर सायकल/स्कूटर, पंखा इत्यादि)		

<table>
<tr><td colspan="2">इस खंड में हम परिवार की प्रवासीय स्थिति के बारे में चर्चा करते हैं कि वे कितनी बार प्रवास करते हैं और कितने सदस्य आजीविका कमाने के लिए अन्य स्थलों पर जाते हैं।</td></tr>
<tr><td>कृपया प्रवासन की स्थिति का वर्णन करें :

क्या आपके परिवार में से कोई भी कमाने के लिए प्रवास पर गया है? आप में से कितने लोग प्रवास करते है? वर्ष की किस अवधि में आप आमतौर पर प्रवास करते है?

रोजगार जो आपको अपने परिवार चलाने के लिए आय का बड़ा हिस्सा प्रदान करता है?</td><td></td></tr>
<tr><td colspan="2">घर की रहने की स्थिति और जीवन शैली निम्नलिखित सवालों से परिलक्षित होती है और इसका तात्पर्य है कि वे इससे कैसे जुड़े हैं।</td></tr>
<tr><td>मैं परिवार की दैनिक जीवन की गतिविधियों और स्थिति जानना चाहूंगी।

घर की संरचना कैसी है? (घर का प्रकार, घर में कमरे, आँगन और मवेशी कोठी)

पीने के पानी का स्रोत कहाँ है? आप इसे कैसे रखते हैं? आप इसे कितनी बार बदलते हैं? क्या आप इसे पीने से पहले इसका शोधन</td><td></td></tr>
</table>

करते हैं?	
खाना बनाने के लिए घर पर किस प्रकार के इंधन का उपयोग किया जाता है?<br><br>आप अपने घर में स्वच्छता कैसे बनाये रखते हैं?<br><br>क्या कूड़ा निस्तारण किया जाता है? आपके पास किस प्रकार का सीवेज सिस्टम है?<br><br>क्या आपके घर में शौचालय की सुविधा है? किस प्रकार का शौचालय है? आपके सफाई करने का तरीका क्या है? आप कहाँ स्नान करते हैं?<br><br>दिन में कितनी बार आप हाथ धोते हैं?	
मैं गांव में परिवार के लिए काम/नौकरी की उपलब्धता जानना चाहती हूँ।<br><br>क्या किसी परिवार के सदस्य को काम की तलाश में प्रवास की आवश्यकता है? यदि हाँ, तो कैसा प्रवास?<br><br>यदि कृषि भूमि है तो	○ आप किस रोजगार में वर्ष के अत्यधिक समय संलग्न रहते हैं?<br>○ क्या साल में किसी भी समय रोजगार के लिए प्रवास होता है?<br>○ कौन प्रवास करता है और कहाँ?<br><br>○ वे किस प्रकार के रोजगार प्राप्त करते हैं?<br>○ कितने दिनों के लिए वे कार्यरत रहते हैं?<br><br>○ एक खेत में किस प्रकार का खाद्यान्य

	फसल होता है?<br>खेते में सिंचाई की सुविधा किस प्रकार से उपलब्ध है?<br>○ परिवार के कितने सदस्य खेत में काम करते है और कितने दिनों के लिए?
यदि पशुधन है तो	○ घर में पशुधन की देखभाल में कितने सदस्य शामिल होते हैं?<br>○ आप पशुधन का प्रबंधन कैसे करते हैं?
यदि वन संग्रह है तो	○ वन संग्रह कार्य में कितने सदस्य शामिल होते हैं?<br>○ वन संग्रह के लिए कौन सा मौसम सर्वाधिक उपयुक्त होता है?
गृह कार्य	○ गृह कार्य में कितने लोग शामिल होते है?<br>○ गृह कार्य में कितना समय लगता है?
इस खंड के प्रश्न आहार की आदतों और प्रथाओं को प्रकट करते हैं। क्योंकि प्रारंभिक वर्षों के दौरान और उसके पश्चात् बच्चे और मां को जो पोषण मिलता है वह कुपोषण का एक महत्वपूर्ण पहलू है।	
मैं एक वर्ष में परिवार की आहार पद्धतियों को जानना चाहूंगी।<br><br>आप सभी ने कल, सुबह से शाम तक क्या–क्या खाया था? भोजन की मात्रा कितनी	

थी? और कितने बार खाया था?<br><br>विशिष्ट अवसरों पर कोई विशेष आहार?<br><br><br><br>आप आमतौर पर बच्चों को भोजन में क्या देते हैं? (मात्रा और आवृत्ति)<br><br><br><br>क्या सभी सदस्यों के लिए समान भोजन परोसा जाता है, या बच्चे को कुछ और दिया जाता है?<br><br><br><br>अपने खाना पकाने के तरीके का उल्लेख करें। (उपयोग से पहले सब्जियों को धोना, हर बार भोजन तैयार करना आदि का वर्णन करें)<br><br><br><br>घर में खाद्य पदार्थों के भंडारण की व्यवस्था कैसी है?<br><br><br><br>वर्ष में किस समय खाद्य पदार्थों को प्राप्त करना मुश्किल है और क्यूं? आप इसका सामना कैसे करते हैं?<br><br><br>बीमारी की अवस्था में बच्चे को किस प्रकार का आहार देते हैं? कितने दिनों के लिए आप उसे विशेष आहार देते हैं?	

<table>
<tr><td>बच्चा कुपोषण का शिकार है कब पता चला?</td><td></td></tr>
<tr><td colspan="2">इस खंड में हम विभिन्न खाद्य और गैर खाद्य पदार्थों, चिकित्सा व्यय पर होने वाले व्यव के बारे में चर्चा करेंगे।</td></tr>
<tr><td>गृह के खर्च में साल भर में विभिन्न खर्च क्या–क्या है?

खाद्य पदार्थों पर दैनिक/साप्ताहिक खर्च

आप कितनी बार राशन खरीदते हैं? इसमें क्या–क्या शामिल है? कितनी राशि का व्यय होता है? (महीना/तीन महीनों में)

गैर खाद्य पदार्थों का ब्यौरा (कपड़ों की खरीदी और खर्च, शिक्षा, यात्रा, एवं त्यौहार में कितनी राशि का व्यय हो जाता है?)

आपने कभी भी खर्च की भरपाई के लिए ऋण लिया है?
उद्देश्य तथा स्रोत का उल्लेख करें?

क्या आपने कहीं से ऋण लिया है? (स्रोत राशि और उद्देश्य निर्दिष्ट करें)</td><td>
<table>
<tr><td>खाद्य पदार्थ का खर्च</td><td></td></tr>
<tr><td>गैर खाद्य पदार्थ का खर्च</td><td></td></tr>
</table>
<table>
<tr><td>मामूली बिमारी (पिछले 15 दिनों में)</td><td>व्यय</td></tr>
<tr><td></td><td></td></tr>
<tr><td>प्रमुख बिमारी (पिछले 15 दिनों में)</td><td>व्यय</td></tr>
<tr><td></td><td></td></tr>
</table>
</td></tr>
</table>

<table>
<tr><td>बच्चों के लिए खाने का खर्च?

क्या आप आपातकालीन स्वास्थ्य स्थिति के लिए कोई बचत कर रहे हैं? बीमारी पर खर्च</td><td><table><tr><td></td><td></td></tr></table></td></tr>
<tr><td colspan="2">सांस्कृतिक प्रथाओं प्रासंगिक हैं क्योंकि वे माता और बच्चे की देखभाल के साथ–साथ आहार प्रथाओं से जुड़े हैं।</td></tr>
<tr><td>मातृ और बाल स्वास्थ्य देखभाल से संबंधित अपनी सांस्कृतिक प्रथाओं का वर्णन करें। (मातृ देखभाल, स्तनपान और बीमारी के दौरान भोजन प्रथाओं और स्वास्थ्य देखभाल उपयोग को कम करना।)

गर्भावस्था और नए बच्चे के जन्म से जुड़े संस्कार क्या हैं?

आपकी शादी कब हुई? आपने पहली बार किस उम्र में गर्भधारण किया था?

क्या आपको गर्भावस्था के दौरान किसी बीमारी का सामना करना पड़ा?

आपका प्रसव किसी अस्पताल या नर्सिंग होम में हुवा था या घर पर? (हाँ या नही) और क्यों?</td><td>.</td></tr>
</table>

क्या आपने अस्पताल में अपना पंजीयन करवाया था? आपके पास प्रसूति पूर्व जाँच करवाया था? (यदि हाँ तो कितनी बार और कहाँ?)	
क्या स्तनपान कराने की प्रथा है? यह किस उम्र तक जारी रहती है आपने स्तनपान कब शुरू किया?	
आप बीमार होने पर अपने बच्चे को स्तनपान कराती हैं?	
आपने किस उम्र में अपने बच्चे को माँ के दूध के अलावा अर्ध ठोस खाद्‌य पदार्थों को खिलाना शुरू किया? कृपया उन वस्तुओं को सूचीबद्ध करें।	
प्रत्येक गर्भावस्था के बीच अंतराल कितना था?	
नवजात शिशु के बीमार पड़ने पर उसे आप पारंपरिक ईलाज जैसे झाड़–फूंक इत्यादी कराते हैं या डॉक्टरों के पास ले जाते हैं? इन दोनो में से आप किसे प्राथमिकता देते हैं और क्यूं?<br>अगर हाँ तो कहाँ उपचार करवाते हैं?	

आपके गाँव के लोग मातृत्व और बाल स्वास्थ्य संबंधी सेवाओं का सामान्य रूप से लाभ कहाँ से उठाते हैं?<br><br>गर्भवती महिलओं और नवजात शिशु की मां को किस तरह का आहार और कितना दिया जाता है?<br><br>क्या आपको लगता है कि आपका बच्चा समान आयु वर्ग के अन्य बच्चों की तुलना में कमजोर है या अक्सर बीमार रहता है?<br><br>आपके परिवार में से कौन–कौन से दैनिक काम में लगे थे जब आप गर्भवती थीं किस महीने तक?<br><br>घर से आपकी अनुपस्थिति के दौरान परिवार के कौन से सदस्य बच्चों को आहार करवाते हैं?<br><br>क्या जन्म, स्तनपान, स्तनपान छुड़ाने और बीमारियों से जुड़ी कोई विशेष मान्यता है?	

यह प्रश्न सार्वजनिक संस्थानों और इसकी सेवाओं के काम के बारे में उत्तरदाताओं की धारणाओं को उजागर करेंगे।	
गाँव में कितनी विभिन्न सार्वजनिक सेवाएं उपलब्ध हैं? इनके क्या–क्या कार्य हैं?<br><br>आपके गाँव में कितने प्रकार की स्वास्थ्य सुविधाएँ उपलब्ध हैं? क्या डॉक्टर पूरे समय उपलब्ध होता है? आपके गाँव में किस प्रकार की मातृ देखभाल संवाएँ उपलब्ध हैं?<br><br>आपके गाँव में कोईं घर पहूँच सुविधा उपलब्ध है?<br>गाँव में कोईं पारंपरिक झाड़–फूंक करने वाला, वैद्य उपलब्ध है?<br><br>क्या आपके गाँव में स्कूल है? वहाँ मध्यान्ह भोजन योजना के अंतर्गत नियमित रूप से भोजन दिया जाता है? शिक्षक नियमित रूप से आते हैं? बच्चा नियमित रूप से स्कूल जाता है?<br><br>क्या आपके गाँव में कोई आंगनबाड़ी है? वह पूरे साल भर खुली रहती है?<br><br>क्या आपका बच्चा आंगनबाड़ी में नामांकित है? क्या आपका बच्चा नियमित रूप से इसमें भाग लेता है?	.<br>.

आंगनबाड़ी केंद्र का समय क्या है? क्या यह समय आपके लिए अनुकूल है?<br><br>आंगनबाड़ी में किस प्रकार का भोजन परोसा जाता है? आपको लगता है कि यह पर्याप्त और पौष्टिक है?<br><br>आपके गाँव से सार्वजनिक वितरण प्रणाली केंद्र कितनी दूर है?<br><br>सार्वजनिक वितरण प्रणाली के तहत किस प्रकार की खाद्य सामग्री प्रदान की जाती है? क्या नियमित रूप से राशन दिया जाता है?<br><br>निकटवर्ती तालुका स्थल तक सड़क और परिवहन सुविधा उपलब्ध है?<br><br>क्या सरकार मनरेगा के तहत रोजगार का कोई अवसर प्रदान करती है? आपके परिवार के कितने सदस्य लगे हुए हैं और कितने दिनों से मजदूरी कर रहे हैं और क्या मजदूरी प्राप्त होती है?<br><br>लोग गाँव में मातृत्व और बाल स्वास्थ्य संबंधी सवाओं का लाभ कहाँ से उठाते है?<br>क्यों :–<br>○ बरसात के मौसम में गाँव में कोई चलित स्वास्थ्य सुविधा उपलब्ध है?	

○ गर्भावस्था के दौरान एएनएम / आशा आपके घर का दौरा करती है? और आपको गर्भावस्था से संबंधित जानकारी, मासिक जाँच का महत्व, गर्भावस्था से संबंधित जटिलता, पोषण का महत्व, स्तनपान का महत्व के बारे में जागरूक करती है?<br><br>ग्राम पंचायत के माध्यम से प्रदान की जाने वाली पानी, स्वच्छता, बिजली, सड़क सेवाएं जो आपको दी जा रही है वह संतोषजनक है?<br><br>मैं परिवार की सांस्कृतिक प्रथाओं को जानना चाहूंगी। एक समुदाय में विभिन्न अवसरों पर विभिन्न अनुष्ठान किए जाते हैं–<br>○ एक घर में अनुष्ठान की प्रथाएँ क्या है?<br>○ दैनिक जीवन में जुड़ी मान्यताएँ क्या है?<br>घर में निर्णय लेने वाला कौन है?	

प्रमुख सूचनाकार साक्षात्कारः–

आंगनबाड़ी कार्यकर्ताओं के प्रश्नः

नाम : आयु / लिंगः

आवासीय पताः सेवा का वर्षः

संस्था का नामः

नामांकित छात्रों की संख्याः नियमित रूप से भाग लेने वाले छात्रों की औसत संख्याः

एस.ए.एम. बच्चों की संख्याः एम.ए.एम. बच्चों की संख्याः

- वर्तमान में आपके केंद्र में कौन से कार्यक्रम चल रहे हैं? क्या माँ और बच्चे के लिए कोई विशेष कार्यक्रम हैं?
- क्या बच्चे और गर्भवती महिलाएँ नियमित रूप से आंगनबाड़ी में भाग ले रहे हैं?
- आंगनबाड़ी में बच्चों को किस तरह का आहार दिया जाता है? मात्रा और आवृत्ति क्या है?
- क्या बच्चे केंद्र में खाना खाते हैं या आप उन्हें इसे वापस घर ले जाने के लिए देते हैं?
- क्या गर्भवती महिलाओं को केंद्र में भोजन उपलब्ध कराया जाता है? यदि हाँ, तो किन–किन खाद्य पदार्थों को किस मात्रा में उपलब्ध कराया जाता है?
- स्वच्छता और भोजन की गुणवत्ता सुनिश्चित करने के लिए आप क्या सावधानी बरतते हैं?
- क्या आप कुपोषण के बारे में जानते हैं? क्या इसके रोकथाम करने के लिए क्या आपके केंद्र में कोई कार्यक्रम चल रहा है?
- आंगनबाड़ी के तहत सेवाएं प्रदान करते समय आपको किन चुनौतियों का सामना करना पड़ता है?
- आंगनबाड़ी में भोजन, धन और कर्मचारियों के वेतन का लाभ उठाते समय आपको किन कठिनाईयों का सामना करना पड़ता है?
- आपके अनुसार बच्चों के बीच कुपोषण की व्यापकता के कारण क्या है? (विशेष रूप से किसी भी मान्यता या प्रथा...) और इससे निपटने के लिए आप क्या तरीके सुझाते है?

सार्वजनिक वितरण प्रणाली आपूर्तिकर्ता के प्रश्नः

नामः आयु / लिंगः

पताः कितने सालों से दुकानः

कार्य करने संस्थानः

वितरण के सरकारी मानदंडः

- आपकी राशन की दुकान से कौन से खाद्य पदार्थ वितरित किए जाते है? किस अंतराल पर राशन वितरित किया जा रहा है? क्या आपूर्ति नियमित है?
- क्या आपको लगता है कि कीमतें उनके खरीदने योग्य हैं और उनके लिए मात्रा पर्याप्त है?
- क्या कोई बाधा है जो वे खाद्य पदार्थों की खरीद में सामना कर रहे हैं? यदि हाँ तो कृपया बताएं।
- क्या आप दुकान के लिए खाद्य पदार्थों की खरीद करते समय किसी समस्या का सामना करते हैं? कृपया समझाएँ।
- आस–पास के गाँवों से राशन की दुकान की दूरी कितनी है? यह कितने गाँवो को राशन प्रदान करता है?
- सबसे दूर का गाँव यहाँ से कितनी दूरी पर है?
- आपके सुझाव के अनुसार वे कौन से खाद्य सामग्री हैं जिसे आप सूची में शामिल करना चाहेंगे।

गैर सरकारी संस्थान के सवालः–

गैर सरकारी संस्था प्रमुख का नामः आयु / लिंगः

पताः स्थापना का वर्षः

संस्था का नामः कितने वर्षों से कार्यरतः

- आपके क्षेत्र में कुपोषण का परिदृश्य क्या है? आपके अनुसार इसके कारण क्या है?
- क्षेत्र में 5 वर्ष से कम आयु के बच्चे किस तरह की बीमारी से पीड़ित है?
- माता और बच्चे की बीमारी से संबंधित क्षेत्र में जनजातियों के बीच स्वास्थ्य सेवाओं के उपयोग करने का क्या तरीका है?
- क्या आपको लगता है कि सार्वजनिक सेवाएँ कुपोषण में महत्वपूर्ण भूमिका निभाती है और कैसे?
- कुपोषण को दूर करने के लिए आपकी संस्था के माध्यम से क्या सेवाएं प्रदान की जाती है?
- आपके क्षेत्र में कुपोषण को कम करने के लिए आपके अनुसार क्या किया जा सकता है?

ए.एन.एम. / आशा के प्रश्नः

नामः आयु/लिंगः

पताः सेवा का वर्षः

संस्था का नामः

- 5 वर्ष से कम आयु की गर्भवती महिलाओं और बच्चों को क्या सेवाएं प्रदान की जाती है?
- सामान्यतः वे किन बीमारियों से पीड़ित रहते हैं?
- स्वास्थ्य सेवाओं का उपयोग कैसे किया जाता है?
- वे किन सामान्य बीमारियों से पीड़ित है?
- स्वास्थ्य सेवाओं का उपयोग कैसे किया जाता है?
- समुदाय को सेवाएं प्रदान करते समय आप किन कठिनाईयों का सामना करते हैं?
- आपके अनुसार समुदाय में कुपोषण क्यों हैं?
- स्वास्थ्य प्रणाली कुपोषण की घटनाओं को कम करने में किस तरह से मदद कर सकती है?

सूचित सहमति

''बालाघाट जिले, मध्यप्रदेश में बैगा जनजाति के बच्चों के बीच कुपोषण के सामाजिक निर्धारकों का अध्ययन करने के हेतू''

शोधकर्ताः पी. शिरिशा

प्रिय उत्तरदाता

नमस्कार,

मैं डॉ. पी. शिरिशा, टाटा इंस्टीट्यूट ऑफ सोशल साइंसेज (टी.आई.एस.एस.) में स्वास्थ्य प्रशासन के स्नातकोत्तर पाठ्यक्रम की छात्रा हूं। स्नातकोत्तर पाठ्यक्रम के अंतर्गत कार्यक्रमए कार्यक्रम को पूरा होने के लिए एक अध्ययन का संचालन करने की आवश्यकता होती है। मैं जनजातीय क्षेत्रों में कुपोषण के सामाजिक निर्धारकों को समझने के बारे में अध्ययन करने का इच्छा रखती हूं। मैंने बालाघाट जिले को अपने अध्ययन क्षेत्र के रूप में चुना है। मैं यह अध्ययन डॉ. नरेंद्र काकड़े, सहायक प्रध्यापक टाटा इंस्टीट्यूट ऑफ सोशल साइंसेज, मुंबई, के मार्गदर्शन में कर रही हूं। इस अध्ययन का मुख्य कार्य उन सामाजिक, आर्थिक और सांस्कृतिक कारकों को समझना है जिनका आदिवासी लोगों के बीच कुपोषण की स्थिति पर प्रभाव पड़ता है।

अध्ययन में भागीदारी पूरी तरह से स्वैच्छिक है। प्रतिभागियों से पूछे गए सवालों के एक सेट के माध्यम से जानकारी एकत्र की जाएगी और प्रतिभागियों द्वारा दी गई प्रतिक्रियाओं को गोपनीय रखा जाएगा और अन्य प्रतिभागियों को भी खुलासा नहीं किया जाएगा, यहां तक कि परिवार के सदस्यों को भी नहीं और इसका उपयोग केवल अनुसंधान उद्देश्य के लिए किया जाएगा। यह अध्ययन प्रतिभागियों को कोई प्रत्यक्ष लाभ नहीं दे सकता है लेकिन यह अध्ययन प्रतिभागियों को किसी प्रकार का कोई नुकसान भी नहीं पहुंचाएगा। उपरोक्त जानकारी पढ़ने के बाद यदि आप अध्ययन में भाग लेने के इच्छुक हैं तो कृपया नीचे हस्ताक्षर करके अपनी सहमति दें।
धन्यवाद!

यदि आपके पास इस अध्ययन या किसी भी चिंता के बारे में कोई प्रश्न हैं तो बेझिझक मुझसे पूछें!

प्रतिवादी का नामः—

प्रतिवादी के हस्ताक्षर / अंगूठा

....
